# Laparoscopic adrenalectomy: Gaining experience by graded approach

**DOI:** 10.4103/0972-9941.26649

**Published:** 2006-06

**Authors:** Abhay N Dalvi, Pinky M Thapar, K Vijay Kumar, Ranjeet S Kamble, Sameer A Rege, Aparna A Deshpande, Nalini S Shah, Padma S Menon

**Affiliations:** Department of General Surgery, Seth G S Medical College & KEM Hospital, Mumbai - 400 012, India; * Department of Endocrinology, Seth G S Medical College & KEM Hospital, Mumbai - 400012, India

**Keywords:** Laparoscopic adrenalectomy, graded approach

## Abstract

**Introduction:**

Laparoscopic adrenalectomy (LA) has become a gold standard in management of most of the adrenal disorders. Though report on the first laparoscopic adrenalectomy dates back to 1992, there is no series of LA reported from India. Starting Feb 2001, a graded approach to LA was undertaken in our center. Till March 2006, a total of 34 laparoscopic adrenalectomies were performed with success.

**Materials and Methods:**

The endocrinology department primarily evaluated all patients. Patients were divided into Group A - unilateral LA and Group B - bilateral LA (BLA). The indications in Group A were pheochromocytoma (n=7), Conn's syndrome (n=3), Cushing's adenoma (n=2), incidentaloma (n=2); and in Group B, Cushing's disease (CD) following failed trans-sphenoid pituitary surgery (n = 8); ectopic ACTH- producing Cushing's syndrome (n=1) and congenital adrenal hyperplasia (CAH) (n=1). The lateral transabdominal route was used.

**Results:**

The age group varied from 12–54 years, with mean age of 28.21 years. Average duration of surgery in Group A was 166.43 min (40–270 min) and 190 min (150–310 min) in Group B. Average blood loss was 136.93 cc (20–400 cc) in Group A and 92.5 cc (40–260 cc) in Group B. There was one conversion in each group. Mean duration of surgical stay was 1.8 days (1–3 days) in Group A and 2.6 days (2–4 days) in Group B. All the patients in both groups were cured of their illness. Three patients in Group B developed Nelson's syndrome. The mean follow up was of 24.16 months (4–61 months).

**Conclusion:**

LA though technically demanding, is feasible and safe. Graded approach to LA is the key to success.

Surgery of the adrenal gland requires knowledge of anatomy, development and physiology of the adrenal glands. The deep retroperitoneal location and close relation to important anatomical structures, made approach to this organ difficult. Adrenalectomy was hence performed by a long abdominal incision with high morbidity.

Laparoscopic adrenalectomy (LA) was first reported in 1992.[1] With technological advancements, the indications have expanded to hypervascular tumors and large-sized benign and malignant adrenal tumors, making laparoscopic adrenalectomy a gold standard in treatment of most adrenal disorders and diseases.[2] Apart from advantages like early recovery, reduced hospital stay and cosmesis, the main benefits of LA over open adrenalectomy are decreased incidence of intra-operative and post-operative hemorrhage, decreased morbidity and mortality.[3,4]

The present prospective study from February 2001 to March 2006, involves data of patients who were subjected to LA at a tertiary referral centre. All the cases were performed using conventional instruments and with the use of electrocautery.

## MATERIALS AND METHODS

This study includes patients subjected to LA from February 2001 to March 2006. Adult patients diagnosed to have adrenal disorder, requiring surgical intervention, were included.

The endocrinology department primarily evaluated these patients. Depending on suspected pathology; serum cortisol, ACTH levels, serum aldosterone and urinary VMA levels were the specific investigations done for diagnosis. Iodine 131 meta-iodobenzy-lguanidine (MIBG) scan was done in patients with pheochromocytoma to rule out multiple and ectopic sites of overproduction. In patients being scheduled for BLA, MRI brain was additionally done in patients with failed trans-sphenoidal surgery, to look for presence of obvious or recurrent pituitary mass.

From the surgical point, contrast-enhanced computerized tomography (CECT scan) or magnetic resonance imaging (MRI) were done in all cases to look for the size of the gland, relation to IVC on right side and renal vein on left side and presence or absence of enlarged lymph nodes. Decision to perform laparoscopic or open adrenalectomy was based on these investigations.

The operating team adopted a graded approach in performing LA. Initially, patients with normal or slightly enlarged glands as in Conn and Cushing were operated, followed then by vascular small pheochromocytoma and finally larger vascular tumors.

Patients who have been excluded till date are those with tumors more than 9 cm in size, suspicion of malignancy, tumor invasion of adjacent organs, recurrent adrenal tumors and patients who were high risk due to cardiopulmonary disease. All the patients who were excluded for laparoscopic approach were operated by an anterior trans-abdominal approach.

The patients were divided into Group A: patients undergoing unilateral LA and Group B: patients requiring BLA.

All these patients were admitted well in advance, till optimum control of blood pressure and blood sugars were achieved. Electrolyte abnormalities were corrected. Patients of pheochromocytoma were maintained on alpha-blockers prior to surgical intervention. Adequate perioperative hydration and steroid cover was given to all patients in Group B.

Patients in Group B were particularly counselled about the disease process and informed about complications of BLA. They were explained about the need for lifelong follow up and were taught about steroid cover during stress.

The data of patient age and sex, side of tumor, indication of surgery, duration of surgery, size of tumor, blood loss, conversion and reason, in-hospital stay and complications were recorded.

All the patients were advised regular follow up and delayed complications or after-effects of adrenalectomy were recorded.

### Technique

All patients were operated under general anesthesia and lateral trans-abdominal flank approach was used. Intra-abdominal pressure was maintained at 12 mm Hg. Electrocautery - predominantly monopolar coagulation and clips were used for hemostasis.

### Right adrenalectomy

**Figure 1:**
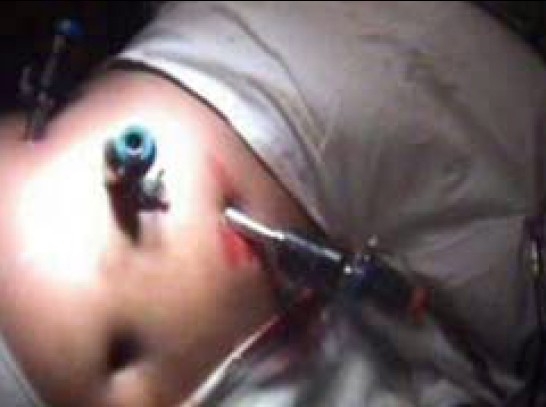
Ports - right adrenalectomy

Four ports were used [Figure 1]. One 10 mm port for a 30-degree telescope and two 10 mm ports for working instruments (sub costal region) were placed in a manner that would render a coaxial vision to the operating surgeon and one 5 mm port at the xiphisternum to aid liver retraction. An additional 5 mm fifth port was inserted if necessary, in the right anterior axillary line to facilitate suction or retraction.

**Figure 2:**
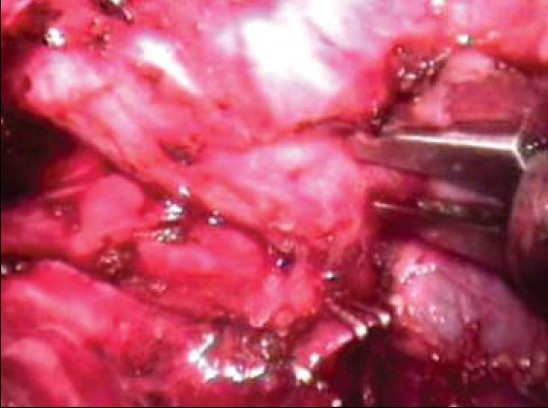
Right adrenal vain

A thin layer of fascia covering the IVC was incised along the right lateral border and the same incision was extended along the peritoneum on the inferior aspect of the liver, laterally upto the right triangular ligament. The latter maneuver aided in additional retraction of the liver and exposure of the gland and the vein. A plane was created between the adrenal gland and the IVC at the upper pole of the kidney and the dissection proceeded cephalad reaching the adrenal vein. The vein was identified on the anterior aspect of the gland, that enters the IVC on its postero-lateral aspect [Figure 2]. After complete dissection of the vein, it was cut between two clips on the patient side and one clip on the specimen side. The gland was then dissected free using a hook with monopolar coagulating current and delivered after placement in the retrieval bag. Ports were closed.

### Left adrenalectomy

**Figure 3:**
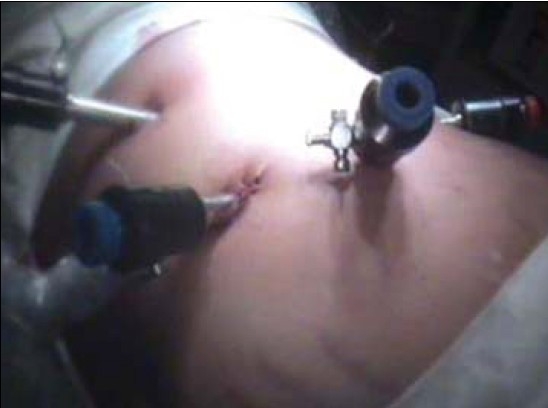
Ports - left adrenalectomy

Three 10 mm sub costal ports, as described for right adrenalectomy were used. The fourth 5 mm port was inserted in the left anterior axillary line to facilitate suction and retraction [Figure 3].

**Figure 4:**
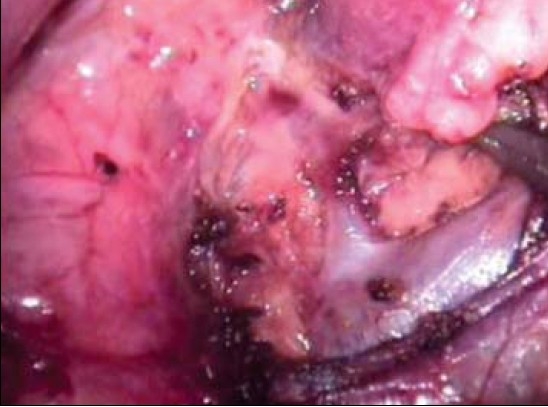
Left adrenal vein

The peritoneum on the lateral aspect of the descending colon was serially incised and the incision extended superiorly to incise the spleno-renal ligament till greater curvature of stomach was seen. This allowed complete retraction of the spleen and the colon by positional gravity and exposed the kidney enveloped in the Gerota's fascia. Dissection was done at the site of the renal hilum, for identification of the renal vein. The adrenal vein was identified along the superior border of the renal vein [Figure 4]. This was clipped and divided. The adrenal gland was then dissected free from the surrounding structures and additional adrenal branches of inferior phrenic vessels were clipped or coagulated. The gland was delivered in a retrieval bag.

Care was taken to remove the intact glands that were sent for histopathological examination. The sheath of the 10 mm ports were closed with 1/0 polyglactin and the skin with 3/0 nylon.

In Group B, right adrenalectomy was performed first, ports were closed and the patient was then repositioned to the opposite lateral decubitus position for left adrenalectomy.

## RESULTS

A total of 24 patients underwent 34 gland excisions from February 2001 to March 2006. Fourteen patients underwent unilateral LA (Group A) and 10 patients underwent BLA-20 adrenal removals (Group B). These included twelve males and twelve females. The age ranged from to 12 to 54 years, with a mean of 28.21 years.

In Group A, 7 patients were operated on the right side and 7 on the left side. The commonest indication was pheochromocytoma (n=7), followed by Conn's syndrome (n=3) and Cushong's adenoma (n=2). Two patients had incidentalomas (one adenomyolipoma and pathology in the other gland could not be determined, as the gland had completely infarcted and developed into an adrenal abscess).

In Group B, the indications for surgery were cushing disease (CD) following failed transphenoidal surgery (n=8), ectopic ACTH producing Cushing's syndrome (n=1) and congenital adrenal hyperplasia (CAH) due to congenital β hydroxylase deficiency (n=1). The patient with ectopic ACTH producing Cushing's syndrome, had been operated for a thymic carcinoid. All investigations to localize the site of overproduction had failed.

Duration of surgery for Group A ranged from 40 to 270 minutes (mean 166.43 min). The duration of surgery in BLA ranged from 150 to 310 minutes (mean 190 minutes). The time taken for change of position in Group B was excluded.

The size of tumor removed in Group A ranged from 2.5 × 3 to 8 × 7 cm, (mean 5 × 4.5 cm) and in Group B ranged from 5 × 2.5 to 7.2 × 4 cm, (mean 5.6 × 3.2 cm).

The blood loss in Group A ranged from 20 to 400 cc (mean 136.93 cc) and in Group B was 40 to 260 cc (mean 92.5 cc). Three patients in Group A required single unit blood transfusion (two patients of pheochromocytoma and one of adenomyolipma with abscess) and none of the patients in Group B required any transfusion.

There were two conversions for bleeding; one in Group A (left pheochromocytoma) and the first patient in Group B on the right side - this patient was a case of ectopic ACTH secreting Cushing's syndrome. After closure of right incision, the left side was laparoscopically completed.

Two patients of Cushing's disease (Group B) and one patient of pheochromocytoma developed wound infection that was treated with antibiotics. One patient of BLA (Congenital Adrenal Hyperplasia) developed hypertensive crisis in the immediate postoperative period and required low dose anti-hypertensive medication. Blood pressure in the rest of the cases was corrected after surgery.

The in-hospital surgical stay in Group A ranged from 1–3 days, with a mean of 1.81 days. In Group B, the same ranged from 2–4 days (average 2.6 days). The patients were transferred to the endocrinology department once on diet. The hospital stay in the endocrinology ward was prolonged in some cases for assessment of blood pressure, sugars and weight in patients with Cushing's disease and due to socioeconomic reasons in other patients.

All patients have been closely followed. The mean follow-up is of 24.16 months (4 months to 61 months). All the patients in Group A are cured and have had no recurrence of disease. In Group B, three patients of CD have developed Nelson's syndrome. Two patients had recurrent pituitary tumor and were subjected to repeat trans-sphenoidal surgery, 12 and 20 months following adrenal surgery. The third patient has only hyper-pigmentation due to elevated ACTH, with no evidence of recurrent pituitary tumor.

## DISCUSSION

The adrenal glands are curious little organs, seated deep in the retroperitoneum amidst important structures. Any abnormality of the functioning of a single or both the glands due to internal or external cause e.g., pituitary gland, can cause havoc in the body physiology. Most of the adrenal abnormalities of size (as in incidentaloma) or of function, require surgical intervention unless contraindicated by patient condition or local invasion of surrounding important structures.

Higashihara[5] in July 1992 and M. Gagner in October 1992[1] are credited with first LA, though it was reportedly first performed by Go *et al* and Suzuki *et al*.[2] There was an initial caution that small glands be removed laparoscopically till adequate experience was reached.[6] Goitein *et al* have estimated the learning curve to be of at least 30 cases to master this technique.[7] It is a coincidence that in the present series, the same strategy of graded approach was employed since February 2001. After a successful excision of the 2.5 cm right adrenal for aldosteronoma, the possibility of performing unilateral adrenalectomy in a progressive manner and bilateral laparoscopic adrenalectomy in a patient of Cushing's syndrome, was explored. Patients with Cushing's syndrome were selected mainly, because there is only hyperplasia, without significant increase in size or vascularity. Gradually, vascular glands as in pheochromocytoma and larger glands as in incidentaloma, were tackled.

Malignant adrenal glands were initially considered as a contraindication for the laparoscopic approach, but adrenal masses upto 13.8 cm without local invasion, have been removed laparoscopically.[8] Sarela *et al* have reported LA for metastatic deposits from the distant primary.[9] Cobb *et al* have also recently reported effective and safe resection of large adrenal malignant tumors. A long-term follow up however, is required to prove its safety in adrenal malignancy.[10] In the present series, no malignant adrenal gland has been tackled.

The contraindications for LA, reported in recent literature are adrenocortical carcinomas that invade surrounding structures, large tumors greater than 10 to 12 cm in diameter, and malignant ACTH-secreting pheochromocytoma with lymphadenopathy.[11] Other limitations to LA are patients unfit for laparoscopy due to coagulation disorder or co-morbid conditions. Previous abdominal scar is no more considered a contraindication for LA.[4]

All the patients in the present series had benign disease. The average size of the gland removed was 5 × 4.5 cm in Group A and 5.6 × 3.2 cm in Group B. Using the graded approach to LA till date, the largest gland removed by us measured 8 × 7 × 6 cm, for a left sided pheochromocytoma. Adrenal glands greater than 9 cm, presence of local lymph nodes or strong suspicion of malignancy, have been operated by open technique. We were encouraged by the success of the graded approach and this prompted the team to embark on larger tumors.

Preoperatively, a surgeon is primarily interested in the size of the gland, its vascularity and proximity or involvement of surrounding important structures. Ultrasonography, CECT scan,[12] MRI[13–15] and MIBG scan[16] are important investigations in localization of adrenal pathology. In the present study, CECT and MRI were relied upon whether to select the patient for laparoscopic approach.

The procedure of LA has been standardized into the retroperitoneal and transperitoneal approach. Miyake *et al* have suggested that the retroperitoneal approach is possibly better in patients with adrenal tumors on the left side with previous abdominal surgery.[17] The retroperitoneal approach[18] has the disadvantage of poor exposure, restricted field and causes problems when complications ensue, specially to a general surgeon. The anterior transperitoneal approach is preferred[11] and user-friendly to surgeons who have been performing open adrenalectomy. Further, laparoscopic skills have been nurtured using the transperitoneal approach in almost all surgical procedures. The same was true in the present study. However, Duh *et al* have reported the outcome of lateral and posterior approach to be similar.[19]

Adrenal vein, being more constant in location, is the most important structure requiring early interruption during adrenalectomy. This is particularly so in secreting tumors, where inadvertent handling of the gland can release hormones in circulation, causing abrupt changes in physiology. The right vein enters the inferior vena cava postero-laterally and is more liable for injury. The arterial supply enters the glands along the periphery and usually consists of small feeders that can be managed by coagulation or clips. The present study followed the same principle of early identification and clipping of the vein with success.

It is important to differentiate the affected adrenal gland from the surrounding fat. This is usually easy due to colour differentiation between the gland and fat, but in cases where the disease evokes a desmoplastic reaction in the surrounding area, the differentiation and dissection can become difficult.[20] In the present study, one patient of Conn's syndrome and one with adenomyolipoma had such a reaction. Once the plane of periadrenal fat is recognized, the patient's body is dissected away from the adrenal gland by patient, gentle and controlled hook dissection. This prevents handling of the gland and consequent release of hormones. With experience and advance in technology like harmonic scalpel, LA is an accepted modality of treatment for most adrenal disorders.[6] Takeda *et al* have reported better dissection of retroperitoneal fat with the use of ultrasonic aspirator.[21] We had no access to advanced gadgets and used only monopolar coagulation current. In our experience, we feel that advanced gadgets are assets, but not an absolute necessity for LA.

A long incision, lower healing power due to increased cortisol levels, increased morbidity and mortality had put the procedure of open bilateral adrenalectomy for CD or Cushing's syndrome into disrepute.[22] BLA has evolved into a life- promising option in this group of patients.[22–25]

In this study, BLA was performed in 10 patients (Group B), as a permanent therapeutic option to correct the symptomatic physiological imbalances [Table 1]. While BLA is an established option in CD following failed trans-sphenoidal surgery,[23,24] it has also been described with success for patients with CAH[26,27] and ectopic ACTH production, where the site of overproduction is not detected.[11] The same was the experience in the present series.

**Table 1:** Physiological corrections after BLA

Symptoms	No. of patients	Corrected
Hypertension	10	9
Diabetes mellitus	4	4
Hypokalemia	5	5
Respiratory distress	3	3
Menstrual irregularities	5	4
Centripetal obesity	7	7
Proximal muscle weakness	7	7
Hirsuitism	8	8
Hyperpigmentation	10	7

The operative time in Group A ranged from 40 to 270 min (mean 166.43 min). This was comparable to that reported by Goitein *et al*[7] (169 min) and Bentrem *et al*[30] (218 min), but more than that reported by Walz *et al*[31] (116 min) and Castilho[32] (107 min). There are reports of simultaneous BLA in literature. In Group B, the operative time ranged from 150 min to 310 min (mean 190 min). The operative time did not include time spent for change of position. Fernandez *et al*[33] (n=15) and Acosta *et al*[25] (n=22) have reported an average operative time of 288 and 240 minutes. respectively. It is not clear but is more likely that the time reported, included the time spent on change of position of the patient. The time taken for BLA is seemingly lower, when compared to time taken while performing unilateral adrenalectomy. This is possibly, because the adrenals removed in Group A were diseased. The adrenals in Cushing's syndrome are normovascular and slightly enlarged. The planes around the adrenal glands are maintained, facilitating faster dissection.

The average blood loss in Group A was 136.93 cc (range 20–400 cc) and in Group B was 92.5 cc (40–260 cc) The reason for increased blood loss in Group A is possibly the same as the increased operative time. Reports from series of laparoscopic adrenalectomies have a reported mean blood loss of 129 cc for unilateral LA, which is comparable with our study and 171 cc for BLA which is higher than the present series.[4] As reported in literature,[3] we experienced the same benefits of LA; only 2 patients of pheochromocytoma and one patient of adrenal abscess required blood transfusion.

There was one conversion, each in both the groups (5.8% in 34 cases), due to bleeding. Various series of unilateral adrenalectomies have reported a conversion rate of 0 to 11%.[31,34,35] Acosta *et al*[25] have reported a 5% conversion rate in his series of BLA (n = 22). The graded approach helped us in keeping the conversion rate comparable to world literature, even in absence of advanced gadgets. It is evident from our results, that all carefully selected patients underwent successful LA, except one conversion in Group B (first patient) and one in Group A (hypervascular pheochromocytoma).

The average in-hospital surgical stay in Group A was 1.81 days. Mean duration of hospital stay in most large series of unilateral adrenalectomy varies between 2.2 to 10.4 days.[31,36] In Group B, mean surgical stay was 2.6 days, with a mean of 3.7 days, as reported in literature.[4]

Intra-operative hemorrhage is the commonest complication described in open and laparoscopic adrenalectomy, accounting for 40% of overall complications.[4] Other complications are bowel injury and pancreatic, splenic, kidney, liver and diaphragmatic injury.[6,7] The overall complication rate in various reports of laparoscopic adrenalectomy is 9.5%.[4] None of the patients in the present series had other major complications, except bleeding in 2 patients who were converted. Three patients (8.82%) developed minor wound infection that was treated with antibiotics.

**Graph 1:**
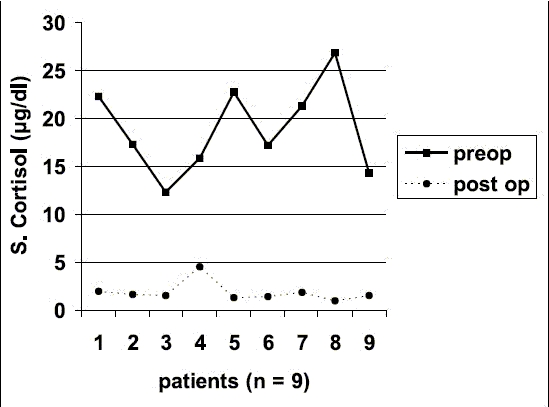
Comparison of pre-operative and post-operative serum cortisol level

The mean follow up in our series is 24.16 months (4–61 months). All patients in Group A are cured. Patients in Group B had excellent response to their symptoms and is similar to that reported by Young *et al*.[11] Negligible post-operative serum cortisol levels confirms cure in these patients. [Graph 1] All the patients in this group are on a maintenance dose of prednisolone 5 mg/day and fludrocortisone 0.05 mg/day. This dose is sufficient for prevention of post-operative adrenal insufficiency.

Nelson's syndrome is a known complication of bilateral adrenal removal. It is characterized by an expanding pituitary lesion with hyper pigmentation, attributed to high levels of ACTH secretion. Symptomatic pituitary tumor warrants excision/ablation.[11] The reported incidence of Nelson's syndrome in literature is 10 – 25%. In the present study, 3 patients developed hyper pigmentation, of which two patients had recurrent pituitary tumor requiring re-do trans sphenoidal surgery, 12 and 20 months following BLA. Third patient has no recurrent tumour and is on regular follow up.

The overall mean mortality of both unilateral and bilateral adrenalectomy is 0.2% (0 – 1.2%).[4] Of these, the majority are in the BLA group. The reasons cited are high anesthesia risk and coexisting medical illness in these patients. There was no mortality in the present study.

Our experience suggests that though LA has a longer learning curve, it is feasible, if careful graded selection of patients is done as in this series.

## CONCLUSION

LA has become a gold standard in the treatment of most adrenal disorders. Knowledge of the anatomy of the adrenal gland and meticulous hemostasis, are key to success in laparoscopic approach.

The popularity of laparoscopic approach for adrenalectomy is mainly due to the magnification and better dissection of this deeply situated gland with lesser complications as compared to highly morbid open surgery requiring long incision. Apart from the conventional advantages of LA over open surgery, the greatest advantage is significantly decreased incidence of intra-operative and post-operative complications, respiratory problems, wound complications like infection and incisional hernias, more so in patients undergoing BLA.

The tip of the telescope becoming the eye of the operating surgeon, reaches close to the gland, giving the surgeon accurate details of the anatomy. Drawing inspiration from this advantage of laparoscopy and adding the graded approach to adrenal surgery, we are of the opinion that LA, though technically demanding, is feasible and safe, even with conventional instruments.
